# Real-World Evidence of Neuropsychiatric Adverse Reactions to Isotretinoin: Insights from EudraVigilance (2005–2025)

**DOI:** 10.3390/ph18091252

**Published:** 2025-08-24

**Authors:** Denisa Viola Szilagyi, Delia Mirela Tit, Claudia Teodora Judea-Pusta, Andrei-Flavius Radu, Gabriela S. Bungau, Ada Radu, Laura Maria Endres, Ruxandra-Cristina Marin

**Affiliations:** 1 Doctoral School of Biomedical Sciences, Faculty of Medicine and Pharmacy, University of Oradea, 410087 Oradea, Romania; szilagyi.denisaviola@student.uoradea.ro (D.V.S.); gbungau@uoradea.ro (G.S.B.); adaradu@uoradea.ro (A.R.); lendres@uoradea.ro (L.M.E.); marin.ruxandracristina@student.uoradea.ro (R.-C.M.); 2 Department of Pharmacy, Faculty of Medicine and Pharmacy, University of Oradea, 410028 Oradea, Romania; 3 Department of Morphology Sciences, University of Oradea, 410087 Oradea, Romania; 4 Department of Psycho-Neurosciences and Recovery, Faculty of Medicine and Pharmacy, University of Oradea, 410073 Oradea, Romania; andreiflavius.radu@uoradea.ro

**Keywords:** isotretinoin, pharmacovigilance, adverse drug reactions, depression, suicide, acne

## Abstract

**Background/Objectives:** Isotretinoin is a highly effective therapy for severe acne, but its potential neuropsychiatric adverse reactions (NPsRs) have been controversial. This study evaluated EudraVigilance data from 2005 to 2025 to better understand the frequency, typology, and predictors of such events. **Methods:** We conducted a retrospective analysis of 33,381 individual case safety reports (ICSRs) related to isotretinoin. Using descriptive statistics, chi-square tests, and logistic regression, we assessed associations between NPsRs and variables such as age, sex, geographic region, and reporter type. **Results:** A total of 9793 cases (29.3%) involved at least one NPsR. Depression (31%) and suicidal ideation (8.6%) were the most frequently reported symptoms. Adolescents (12–17 years) had the highest proportion of NPsR cases, while male patients and reports submitted by non-healthcare professionals were significantly overrepresented. Reports from non-European Economic Area countries also had slightly increased odds of including NPsRs. All predictors were statistically significant in the logistic regression model, though the explained variance was modest (Nagelkerke R^2^ = 0.065). **Conclusions:** Neuropsychiatric reactions remain a prominent and persistent signal in isotretinoin pharmacovigilance, particularly among younger patients and non-professional reporters. Although causality cannot be inferred from spontaneous reporting data and confounding factors like acne-related depression cannot be excluded, these findings highlight the clinical value of pre-treatment psychiatric screening, patient-centered education, and proactive mental health monitoring throughout isotretinoin therapy.

## 1. Introduction

Acne vulgaris is one of the most common dermatological conditions globally, characterized as a chronic inflammatory skin disorder that typically affects the pilosebaceous units. It poses a substantial burden, particularly among adolescents and young adults, due to its persistence and psychosocial impact [1]. Many endogenous and exogenous factors contribute to the etiology of acne. Hormones released by the adrenal gland, ovaries, and pancreas, as well as inflammatory processes from various sources and stress, can all trigger sebum oxidation, which may be related to the development of acne. The first category implies endocrine disorders, chronic stimulation of immune systems, and genetic predispositions, whereas the latter can be due to cosmetics, stress, and tobacco [2]. Moreover, building on this multifactorial framework, a study showed that the complex relationship between metabolic preconditioning, circulating levels of glutathione peroxidase, and catecholamines could represent a potential factor in both the appearance and the progression in severity of acne [3].

Recent data from the Global Burden of Disease Study 2021 indicate that the age-standardized prevalence rate of acne vulgaris among adolescents and young adults increased from 8563.4 per 100,000 population in 1990 to 9790.5 per 100,000 in 2021, reflecting a significant upward trend over the past three decades. This increase underscores the growing burden of acne vulgaris globally, particularly among adolescents aged 15–19 years, who exhibit the highest age-specific prevalence, with a rate up to 90%. However, the condition can affect adults too [4,5]. Thus, with an estimated worldwide incidence (for all ages) of 9.38%, acne vulgaris is the eighth most common disease globally [6]. These statistics underscore the importance of effective therapeutic strategies, particularly as the global impact of acne continues to escalate. The management of acne vulgaris follows a severity-based, stepwise approach. For mild to moderate disease, first-line treatment usually includes topical agents like benzoyl peroxide, retinoids (e.g., adapalene or tretinoin), and antibiotics that address the pathogenic factors of follicular hyper keratinization, microbial colonization, and inflammation [7].

When acne progresses to a moderate or more extensive form, systemic therapies become necessary; oral tetracyclines (notably doxycycline and minocycline) are commonly prescribed, and in female patients with features of hyperandrogenism, hormonal treatments, including combined oral contraceptives and spironolactone, offer additional benefit by modulating androgen-driven sebum production [7,8].

For patients with severe nodular acne, significant scarring, or disease refractory to both topical and systemic antibiotics, the systemic retinoid isotretinoin is the treatment of choice [7]. Isotretinoin’s mechanism addresses all major aspects of acne pathophysiology, normalizing follicular keratinization, reducing sebum production, inhibiting *Propionibacterium acnes* proliferation, and exerting anti-inflammatory effects, and has demonstrated the ability to induce prolonged remission in the majority of treated patients [9]. The recommended initiation dose is 0.5 mg/kg/day (oral administration), with the option to escalate to 1.0 mg/kg/day depending on patient tolerance and clinical response, aiming for a cumulative dose that maximizes long-term efficacy [10].

Isotretinoin revolutionized the treatment of severe acne vulgaris by inducing prolonged remissions in resistant cases. Nevertheless, despite its usefulness since its introduction in 1982, isotretinoin’s safety profile has been closely examined due to several serious adverse effects [11]. Mucocutaneous symptoms such as cheilitis and xerosis are common, serum lipids and liver enzymes must be checked periodically to detect hyperlipidemia and hepatotoxicity, and, critically, strict pregnancy prevention protocols are required for women of childbearing potential given the drug’s high teratogenic risk [12]. But one of the most important concerns for clinicians is linked to its possible neuropsychiatric responses. The package insert for isotretinoin was modified in 1998 to add warnings concerning the risk of depression and suicidality [13].

Reports have shown that isotretinoin users have experienced sadness, mood changes, and, in rare circumstances, suicidal thinking and behavior. These high-profile reports in isotretinoin users have periodically circulated in the media, raising public and professional concern and even prompting litigation [14]. In terms of biological plausibility, several hypotheses have been proposed to explain how isotretinoin could influence the central nervous system. Retinoids are known to have neuroactive properties; isotretinoin can cross the blood–brain barrier and may interact with retinoid receptors in the brain [15].

Animal studies have shown that isotretinoin can reduce hippocampal neurogenesis and alter serotonin signaling in rodents, changes that could theoretically underpin depressive behavior [16]. Additionally, isotretinoin has been linked to hyperhomocysteinemia and reductions in folate and vitamin B12 levels in patients. Elevated homocysteine is a risk factor for depression and cognitive dysfunction [17].

Some researchers have speculated that isotretinoin-induced metabolic changes (like vitamin B12/folate deficiency) might contribute to neuropsychiatric side effects, as supplementation with these vitamins has been reported to ameliorate depressive symptoms in certain isotretinoin patients [18]. Despite these reports, establishing a causal link between isotretinoin and psychiatric disorders has proven challenging. Acne itself is associated with an increased risk of depression and suicidal ideation independent of treatment [19], making it difficult to separate drug effects from the background psychological burden of the disease. Thus, individualized clinical judgment becomes crucial. Ensuring a thorough evaluation of each patient’s prior treatment history, comorbidities, and risk factors allows clinicians to balance isotretinoin’s profound benefits against its safety considerations, in full alignment with contemporary best-practice guidelines [20].

Despite these precautionary measures, ongoing research has sought to clarify the psychiatric safety profile of isotretinoin through large-scale observational and epidemiological analyses [21]. However, research on this topic has yielded mixed findings. Epidemiological studies and meta-analyses have not conclusively demonstrated an excess risk of psychiatric disorders due to isotretinoin. For example, a large Taiwanese cohort study (n = 29,943) found no increased incidence of psychiatric diagnoses among isotretinoin-treated patients compared to untreated acne patients, even at higher doses or longer treatment durations [22]. Similarly, a cohort study, using global health records, observed that isotretinoin users did not have higher odds of depression, anxiety, or self-harm than patients on other acne therapies; notably, isotretinoin was associated with lower risk of psychiatric outcomes than oral antibiotics in that analysis [23].

Given this context, post-marketing pharmacovigilance data can provide valuable real-world evidence to better understand the scope and nature of neuropsychiatric adverse reactions reported during isotretinoin therapy. The European Medicines Agency’s (EMA) EudraVigilance database [24] proved to be useful in tracking such adverse drug reactions (ADRs). Analyses of this database have underlined the need for pharmacovigilance in recognizing and understanding isotretinoin’s risk profile, particularly in terms of neuropsychiatric consequences [25]. Recognizing the value of post-marketing surveillance, the present study aims to leverage 20 years of real-world adverse event reports from the European Medicines Agency’s EudraVigilance database for identifying and characterizing the neuropsychiatric adverse reactions associated with isotretinoin. The use of large real-world data analytics, much like the computational intelligence approaches applied in diagnostic systems for neuropsychiatric outcomes in other medical domains [26], underscores the increasing trend toward advanced algorithmic methods in pharmacovigilance. Moreover, given that demographic factors such as educational level can meaningfully influence both disease reporting and health outcomes [27], our analysis considered demographic and reporting biases to inform more accurate risk interpretation.

By analyzing a large dataset of spontaneous reports (2005–2025), we seek to (1) determine the frequency and types of neuropsychiatric events reported with isotretinoin, and (2) examine demographic or reporting factors associated with these events. Moreover, our aim was to provide an up-to-date safety profile based on real-world reporting trends, thereby informing clinicians and regulators on potential risks and guiding patient monitoring strategies.

## 2. Results

### 2.1. Descriptive Overview

Over the 20-year pharmacovigilance period (2005–2025), a total of 33,381 individual case safety reports (ICSRs) related to isotretinoin were documented in the EudraVigilance database. Table 1 provides a comprehensive overview of the demographic profile of patients, the nature of reporters, and the geographic origin of these reports.

With respect to patient sex, slightly more than half of all cases involved female patients (50.4%, n = 16,806), while 43.9% (n = 14,670) concerned male patients. In a minority of cases (5.7%, n = 1905), sex was not reported. This distribution reflects the known clinical indication of isotretinoin for severe acne, a condition affecting both sexes, although with differing treatment-seeking behaviors and clinical presentations. In terms of age distribution, most cases were concentrated among adolescents and young adults, the primary treatment population for isotretinoin. Adults aged 18–64 years accounted for 49.2% (n = 16,428) of reports, followed by adolescents aged 12–17 years (22.5%, n = 7512). Reports involving pediatric patients under 12 years of age were rare (<1% combined), and very few cases were recorded among older adults aged 65 years and above. Of note, 26.7% (n = 8919) of cases lacked age specification, which may reflect incomplete reporting or challenges in data entry across jurisdictions.

Regarding the type of reporter, most ICSRs were submitted by healthcare professionals, including physicians, pharmacists, and clinical staff, representing 58.7% of reports (n = 19,596). However, a substantial proportion of cases (41.0%, n = 13,690) were reported by non-healthcare professionals, such as patients or caregivers. In 0.3% of cases (n = 95), the identity of the reporter was not specified. From a geographic perspective, the majority of reports (62.6%, n = 20,886) originated from non-European Economic Area (non-EEA) countries, while the EEA accounted for 37.4% (n = 12,494). Only a single report lacked geographic information.

**Figure 1:**
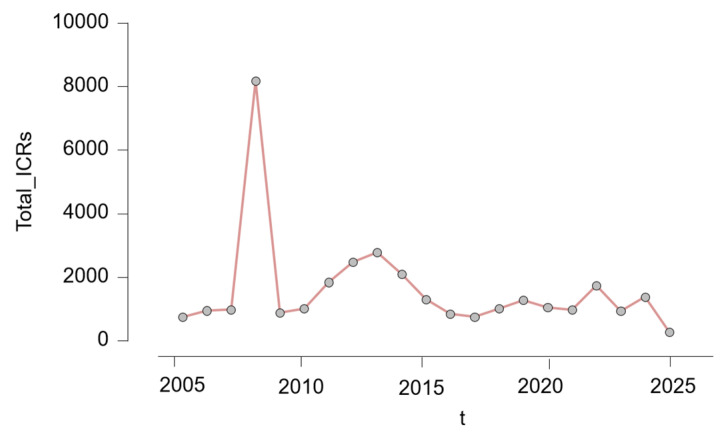
Annual number of individual case safety reports (ICSRs) involving isotretinoin (2005–2025); t = year.

The number of ICSRs varied considerably across the 20-year observation period. As illustrated in Figure 1, reporting volumes increased sharply after 2007, reaching a dramatic peak in 2008, with over 8000 cases submitted in a single year. Following this peak, annual reporting stabilized at more moderate levels, generally ranging between 700 and 2800 reports per year. Another period of relatively high reporting occurred between 2011 and 2015. In more recent years, from 2020 onward, reporting volumes showed a slight decline, possibly influenced by external factors such as the COVID-19 pandemic, shifting public health priorities, or changes in isotretinoin prescription patterns.

### 2.2. Neuropsychiatric Adverse Reactions

#### 2.2.1. Overview and Frequency

Out of the 33,381 isotretinoin-related ICSRs recorded between 2005 and 2025, a total of 9793 cases (29.3%) included at least one neuropsychiatric adverse reaction (NPsR), encompassing both psychiatric events (such as depression, suicidal ideation, and anxiety) and neurological symptoms (such as headache and dizziness), based on MedDRA Preferred Term classification. The annual proportion of NPsRs fluctuated considerably, ranging from a low of 12.7% in 2022 to a peak of 41.7% in 2012, with an average proportion of approximately 29.3% across the entire study period. The highest absolute number of ICSRs was reported in 2008 (n = 8178), of which 32.0% involved neuropsychiatric components. The interval between 2011 and 2015 was marked by consistently elevated proportions of neuropsychiatric reports. From 2022 onward, a continued decline in the proportion of neuropsychiatric reports was observed. In 2022, such reactions accounted for only 12.7% of all cases, and this decreasing trend persisted through 2025.

The observed fluctuations in reporting rates were statistically significant, as confirmed by a chi-squared test for independence (χ^2^ = 928.94, df = 20, *p* < 0.001), indicating that the variations across years were unlikely to be due to chance alone. A detailed year-by-year breakdown of NPsR versus non-NPsR cases is provided in Table 2, highlighting both absolute counts and relative proportions over time.

**Figure 2:**
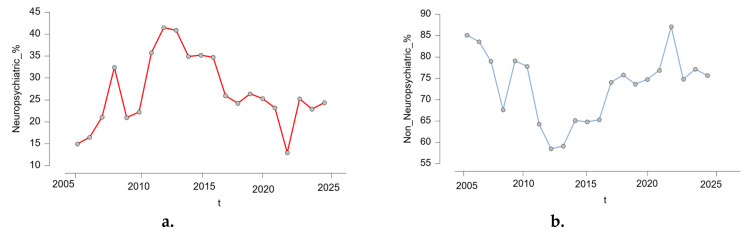
Yearly distribution of neuropsychiatric vs. non-neuropsychiatric adverse reactions to isotretinoin (2005–2025) (**a**). Neuropsychiatric adverse reactions, (**b**). non-neuropsychiatric adverse reactions; t = year.

Figure 2 shows the yearly evolution of the proportion of reported NPsRs and non-neuropsychiatric ARs from 2005 to 2025. Panel (a) displays the percentage of non-neuropsychiatric ARs among all isotretinoin-related cases, while panel (b) shows the corresponding percentage for NPsRs. The two trends appear inversely correlated across time, with NPsRs peaking between 2011 and 2013, reaching over 41% in 2012, followed by a gradual decline, while non-NPsRs showed a complementary upward trend. This comparative presentation highlights the changing pattern of reporting priorities or possible shifts in awareness over the study period. The proportion of NPsRs increased notably until its peak in 2012–2013, followed by a moderate and consistent decline. In contrast, non-NPsRs followed an inverse pattern, with lower percentages during the peak years of NPsR reporting and higher proportions in the subsequent years.

This visualization illustrates dynamic shifts in reporting emphasis, which may reflect increased public or clinical awareness of psychiatric risks associated with isotretinoin during certain periods, changes in pharmacovigilance practices, or external influences such as regulatory alerts and media coverage.

#### 2.2.2. Typology of Neuropsychiatric Reactions

A total of 16,049 NPsRs were identified across 9793 individual cases, underscoring the frequent occurrence of multiple neuropsychiatric events within the same patient report. On average, each case involving neuropsychiatric symptoms included approximately 1.61 reactions, reflecting the clinical complexity of these presentations.

The vast majority of reported neuropsychiatric adverse reactions (NPsRs) were psychiatric in nature, consistent with isotretinoin’s known association with mood and behavioral disturbances. The most frequently reported adverse reaction was depression, accounting for 4967 occurrences, or 30.67% of all neuropsychiatric reactions. This symptom alone was reported in over half of all cases that included at least one NPsR (50.72% of cases). Other commonly reported psychiatric events included suicidal ideation (n = 1348; 8.32% of reactions; 13.76% of cases), anxiety (n = 1092; 6.74%; 11.15%), and emotional distress (n = 780; 4.82%; 7.96%). Reports of headache (n = 1200; 7.41% of reactions) were the most prominent among neurologic symptoms, often appearing in combination with psychiatric complaints.

Other notable events included suicide attempt (n = 445; 2.75% of reactions; 4.54% of cases), as well as depressed mood (n = 346; 2.14%; 3.53%) and completed suicide (n = 335; 2.07%; 3.42%). Among neurologic presentations, dizziness (n = 314; 1.94%) and seizure (n = 281; 1.73%) were the most frequently reported, further highlighting the diverse neuropsychiatric profile of isotretinoin-related adverse events. Table 3 presents the most commonly reported NPsR, based on MedDRA preferred terms, with corresponding frequencies and relative proportions across the dataset.

When stratified by age and sex, the majority of neuropsychiatric adverse reactions were reported in adolescents (12–17 years) and adults (18–64 years). In adolescents, depression was the most frequent event, accounting for 37.4% of cases in females (n = 483) and 62.3% in males (n = 806). Suicidal ideation showed a similar sex imbalance, with 69.7% of adolescent cases occurring in males (n = 272) compared to 29.5% in females (n = 115). Suicide attempt (58.5% males vs. 40.5% females) and completed suicide (80.9% males vs. 18.0% females) were also more common in adolescent males, highlighting their particular vulnerability to suicidality.

Among adults, depression predominated in both sexes but was more frequently reported in women (58.1%, n = 1641) than men (41.6%, n = 1176). Headache (68.2% females vs. 31.2% males) and anxiety (57.9% females vs. 41.8% males) also showed a female predominance. By contrast, emotional distress (53.3% males vs. 46.3% females) and seizure (51.7% males vs. 47.4% females) were somewhat more frequent among men.

Reports in children ≤11 years were rare (<1% of all events), and those in neonates (0–1 month) and infants (2 months–2 years) likely reflected transplacental exposure. Older adults (≥65 years) contributed only sporadic cases across both sexes. Notably, a substantial proportion of events occurred in patients with unspecified age or sex, particularly for depression (n = 827, 49.9%) and suicidal ideation (n = 280, 45.7%). The distribution of top reported NPsRs by age and sex is presented in Table 4.

### 2.3. Non-Neuropsychiatric Adverse Reactions

Among the 23,588 isotretinoin-related ICSRs without any neuropsychiatric component, the most frequently reported non-neuropsychiatric adverse reactions were gastrointestinal, dermatological, and musculoskeletal in nature (Table 5). Inflammatory bowel disease (IBD), encompassing Crohn’s disease and ulcerative colitis, emerged as the leading category, reported in 15.4% of all non-NPsR cases. Dermatological events were also common, with dry skin and lip dryness (cheilitis). Musculoskeletal complaints, including arthralgia, were reported in over 2% of non-NPsR cases. Metabolic laboratory abnormalities such as elevated blood triglycerides were also frequent.

### 2.4. Comparative Analysis: Neuropsychiatric vs. Non-Neuropsychiatric Cases

To better understand the contextual and demographic factors associated with NPsRs, we conducted a comparative analysis between cases that reported at least one NPsR and those that did not. Statistically significant differences emerged across all examined variables, including age group, sex, reporter type, and geographic origin (all *p*-values < 0.001, Table 6).

Age was a major determinant. Adolescents aged 12–17 years showed the highest proportion of neuropsychiatric reports (36.3%) relative to their total case count, followed by adults aged 18–64 years (32.2%). In contrast, infants under 1 month of age and children aged 2 months to 2 years had the lowest proportions of NPsRs (4.6% and 5.8%, respectively), likely due to minimal exposure to isotretinoin in these age groups. These consisted mainly of unspecific terms such as anxiety and insomnia, as well as occasional seizure events. Such reports are most likely attributable to indirect exposure (e.g., transplacental transfer or breastfeeding) and to caregiver or clinician interpretation, rather than reflecting primary psychiatric manifestations in this age group.

Sex-based differences were also notable. While females represented a slightly higher total number of cases overall, males had a greater proportion of neuropsychiatric reports (32.6%) compared to females (28.1%). Reports missing sex information exhibited a markedly lower proportion of NPsRs (15.1%), suggesting potential underreporting or data completeness bias in this subgroup.

Reporter type significantly influenced the reporting of neuropsychiatric symptoms. Reports submitted by non-healthcare professionals (e.g., patients or caregivers) were associated with the highest proportion of NPsRs (38.9%), whereas those from healthcare professionals had a significantly lower share (24.0%). This discrepancy likely reflects differences in perception, reporting thresholds, or recognition of psychiatric symptoms between professional and non-professional reporters.

Geographic origin also played a role. Reports from outside the EEA had a higher proportion of NPsRs (31.3%) than those from EEA countries (26.4%). This may reflect regional disparities in pharmacovigilance practices, access to psychiatric care, or cultural differences in the recognition and documentation of mental health symptoms.

### 2.5. Associations Between Demographic and Reporting Factors and Neuropsychiatric Adverse Events

To explore independent predictors of NPsR reporting in isotretinoin cases, we performed a multivariable logistic regression analysis. The binary outcome was defined as the presence (1) or absence (0) of at least one neuropsychiatric adverse reaction per case. The final model (M_1_) included four categorical predictors: patient age group, sex, reporter type, and geographic region (EEA vs. non-EEA). For regression purposes, the detailed age categories presented in Table 7 were aggregated into broader groups (<12 years, 12–17 years, 18–64 years, ≥65 years, and NSp) to ensure adequate sample sizes and model stability.

Compared to adolescents aged 12–17 years, the group with the highest proportion of neuropsychiatric reports, patients under 12, had substantially lower odds of experiencing neuropsychiatric reactions (OR = 0.23, 95% CI: 0.17–0.32, *p* < 0.001). Similarly, adults aged 18–64 showed modestly reduced odds (OR = 0.82, *p* < 0.001), and individuals aged ≥ 65 years also had a significantly lower likelihood (OR = 0.58, *p* = 0.009). Reports with unspecified age were less likely to include NPsRs (OR = 0.44, *p* < 0.001), though interpretation is limited by missing data.

Regarding sex, male patients had slightly higher odds of neuropsychiatric reporting compared to females (OR = 1.11, 95% CI: 1.06–1.17, *p* < 0.001), while reports with unspecified sex were less likely to involve such reactions (OR = 0.77, *p* < 0.001).

Reporter type emerged as one of the strongest predictors. Cases submitted by non-healthcare professionals, most often patients or their family members, were nearly twice as likely to include neuropsychiatric symptoms compared to those submitted by healthcare professionals (OR = 1.83, *p* < 0.001). This finding may reflect differences in symptom perception or reporting behavior across professional and non-professional sources.

Geographic region was also a significant predictor. Reports originating from outside the European Economic Area (non-EEA) were modestly more likely to include neuropsychiatric adverse reactions than those from EEA countries (OR = 1.16, *p* < 0.001).

The regression results, including odds ratios and confidence intervals for each predictor, are detailed in Table 7**.** The full model significantly improved prediction compared to the null model (ΔΧ^2^ = 1553.26, *p* < 0.001). Although the explained variance was modest (Nagelkerke R^2^ = 0.065), these results are consistent with expectations for pharmacovigilance datasets, where many relevant clinical and contextual variables may be unreported.

**Figure 3:**
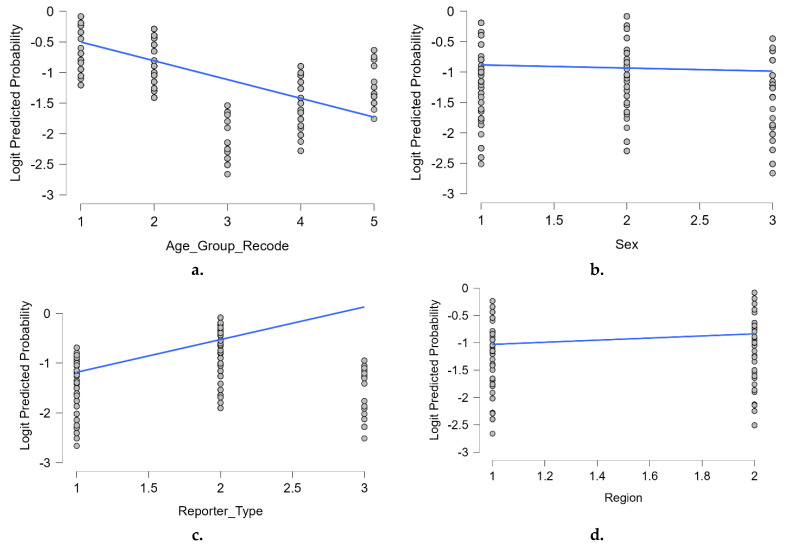
Predicted logit probabilities by key predictors: (**a**). age group (recode); (**b**). sex; (**c**). reporter type; (**d**). geographic region. Blue lines indicate the fitted linear trend within each category. The values were derived from the final multivariable logistic regression model. Predictors were coded as categorical variables with reference groups used in the regression model: age_group_recode = 1: 12–17 years ( reference group.), 2: 18–64 years, 3: ≥65 years, 4: <12 years, 5: not specified sex = 1: female ( Reference group), 2: male, 3: not specified; reporter_type = 1: healthcare professional (reference group.), 2: non-healthcare professional, 3: not specified region = 1: EEA (reference group), 2: non-EEA.

To further illustrate the associations identified in the multivariable logistic regression model, we plotted the predicted logit probabilities of neuropsychiatric adverse reactions by each of the key categorical predictors included in the model. Figure 3 provides a visual representation of the relationship between each predictor and the estimated likelihood of reporting a neuropsychiatric reaction. Panel **a.** highlights a clear negative trend with age, indicating a decreasing likelihood of neuropsychiatric reports among older age groups. In panel **b.**, the probability appears slightly higher in male patients compared to females, consistent with the modest but significant positive association seen in the model. Panel **c.** shows a steeper positive slope, reflecting the substantially higher odds of neuropsychiatric reactions reported by non-healthcare professionals. Finally, panel **d.** depicts a modest increase in predicted probability for reports originating from non-EEA regions compared to EEA countries.

## 3. Discussion

### 3.1. Interpretation of Findings in the Context of Existing Literature

Acne vulgaris is a common dermatological condition, particularly affecting adolescents and young adults. Isotretinoin remains the most effective treatment for moderate to severe acne, offering sustained remission. However, its use is accompanied by a well-documented spectrum of adverse effects, ranging from mucocutaneous dryness to serious concerns such as teratogenicity and potential neuropsychiatric risks [11]. These latter effects have generated ongoing debate and regulatory attention, especially considering their implications for vulnerable populations. In this context, post-marketing surveillance through pharmacovigilance systems plays a crucial role in identifying adverse drug reaction (ADR) signals and guiding evidence-based policy. Systematic evaluation of spontaneous reporting data is therefore essential for understanding the characteristics and scope of neuropsychiatric events potentially associated with isotretinoin use.

The present analysis of 33,381 isotretinoin-associated ICSRs provides several noteworthy observations. One of the most striking findings is the sharp increase in reporting observed in 2008, with over 8000 reports submitted that year, far above the approximate annual average of 1000. The period coincided with major regulatory and public awareness events. In the United States, the iPLEDGE risk management program became mandatory in March 2006, imposing stringent prescribing, dispensing, and monitoring requirements to prevent isotretinoin-related teratogenicity [28]. That year, the U.S. FDA updated the Accutane^®^ label to prominently warn about depression, psychosis, suicidal ideation, suicide attempts, and completed suicide, while reinforcing iPLEDGE protocols [29].

In Europe, regulatory actions in late 2007 strengthened psychiatric safety messaging. The French medicines agency issued a safety communication on 22 November 2007 advising clinicians and patients about possible depression and suicidal behavior [30], while the EMA retinoid referral required oral retinoid leaflets to include depression/anxiety warnings and specific monitoring advice [31].

In Australia, October 2008 scheduling records explicitly advised monitoring for depression in isotretinoin users [32]. This period also saw heightened clinical attention, including a case-crossover study reporting a significant association between isotretinoin and depression [28], as well as sustained scholarly debate in 2009–2010 supported by national cohort data from Sweden [33]. Furthermore, French parliamentary records note that reports of new suicide cases in 2008 prompted the creation of a national working group [34].

Although no formal study has quantified isotretinoin-specific media coverage in 2008, high-profile reports in the UK, France, and Australia on alleged suicides among adolescents likely amplified reporting. Such “notoriety effects,” where intense public or professional attention to a drug-adverse reaction pair temporarily increases spontaneous reports without altering true incidence, are well documented in pharmacovigilance [11,35].

Within isotretinoin literature, increased psychiatric ADR reporting has been observed following high-profile safety communications, consistent with our interpretation.

Pharmacovigilance data from the iPLEDGE era (2006–2008) further show that while total prescribing declined, the proportion of prescriptions with documented contraceptive use rose, reflecting altered monitoring and reporting behaviors [36]. Together, these isotretinoin-specific regulatory milestones, safety communications, media coverage, and professional debate support the conclusion that the 2008 peak reflects stimulated reporting and retrospective data entry within an evolving pharmacovigilance landscape, not a verified increase in true event rates [11,21].

Temporal fluctuations were also found in other pharmacovigilance databases, often reflecting changes in surveillance intensity or public awareness. Although recent FAERS analyses confirm a strong signal for early-onset neuropsychiatric adverse events, including suicidal ideation, they do not report year-specific reporting spikes. Therefore, it is likely that the 2008 peak in our dataset reflects heightened reporting activity rather than a sudden increase in the underlying clinical burden [37,38].

Beyond temporal trends, the demographic distribution of the reported cases offers valuable insight. In our dataset, over half of the cases involved female patients (50.4%), and nearly three-quarters (71.7%) were adolescents or young adults, populations that represent the typical therapeutic target group for isotretinoin. These findings are consistent with other studies and reaffirm the expected demographic profile of isotretinoin users. However, a focused analysis of 2839 isotretinoin-related suicide and suicide-related reports from the FAERS database revealed a slightly different distribution, with a higher proportion of male patients (56.9%) compared to females (43.1%), potentially indicating differential vulnerability or reporting patterns by sex in psychiatric outcomes [39].

In terms of geographic origin, most reports in our study originated from outside the European Economic Area (62.6%), reflecting EudraVigilance’s broader international reach through data-sharing mechanisms. This aligns with the EMA’s increasing role in capturing global pharmacovigilance data and supports the generalizability of findings across healthcare contexts. Similarly, the distribution of reporter types followed expected pharmacovigilance trends, with 58.7% of cases submitted by healthcare professionals. Notably, non-healthcare reporters (41.0%) also contributed a substantial share, especially in cases involving neuropsychiatric symptoms. This supports previous findings suggesting that patient-reported outcomes may be particularly valuable in detecting subjective or underrecognized adverse reactions such as mood changes, suicidality, or anxiety [37,40].

Our results showed that nearly one-third of all spontaneous isotretinoin-related adverse event reports between 2005 and 2025 included at least one neuropsychiatric symptom (29.3%), underscoring the prominence of mental health concerns in isotretinoin pharmacovigilance. This proportion is comparable to the global prevalence of depression (22%), anxiety (29%), and suicidal thoughts (12%) reported in untreated acne cohorts, as recently summarized in a meta-analysis of 43 studies spanning 1961–2023 [41], placing our findings at the upper range of expected psychiatric comorbidity in this population. Depression was the most reported term, comprising 31% of all neuropsychiatric events and present in over half of neuropsychiatric cases. Suicidal ideation was also prominent (8.4% of reactions; 13.6% of cases). These patterns are consistent with previous U.S. studies, despite differences in reporting systems, underscoring the validity of these signals across international datasets. When contextualized against European epidemiological data, the signal remains noteworthy. Between 2018 and 2020, depressive symptoms affected 6.54% of adults across Europe, while lifetime prevalence of suicidal ideation and suicide attempts were estimated at 3.9% and 0.8%, respectively [42]. Additionally, Eurostat data from 2021 documented 47,346 completed suicides across EU countries, corresponding to a rate of 10.2 per 100,000 inhabitants [43]. While the absolute number of isotretinoin-linked neuropsychiatric reports appears modest, the relative burden of these reactions, especially when considered against spontaneous reporting systems, remains consistent with broader mental health epidemiology, justifying continued vigilance [44].

Transatlantic data converge on similar findings. A FAERS analysis (1997–2017) identified depressive disorders (42%), emotional lability (17%), and anxiety (14%) as leading psychiatric ADRs. In total, 2278 cases of suicidal ideation, 602 suicide attempts, and 368 completed suicides were reported in relation to isotretinoin. Intriguingly, the overall incidence of suicidal behavior was lower than in the general U.S. population, suggesting that acne-related psychosocial burden may partially confound these associations [14]. These findings reinforce the need to interpret spontaneous reports within the broader psychosocial context of acne, especially in adolescents, a group inherently at elevated risk for mood disorders [45]. Consistently, our stratified analysis showed that adolescent males accounted for nearly 70% of suicidal ideation, 59% of suicide attempts, and more than 80% of completed suicides. In contrast, adult females were disproportionately affected by depression (58%), headache (68%), and anxiety (58%). Such age- and sex-specific patterns emphasize the need for tailored vigilance and targeted risk communication.

Beyond neuropsychiatric outcomes, several non-NPsRs were frequently reported. Among these, gastrointestinal disorders were prominent, with inflammatory bowel disease (IBD) accounting for 15.4% of non-NPsR cases and irritable bowel syndrome (IBS) for 2.23%. While the causal link between isotretinoin and IBD remains controversial, several studies have suggested a possible association with ulcerative colitis in susceptible individuals [46]. Mucocutaneous effects were also common, notably dry skin (3.36%) and cheilitis (2.67%), which are well documented as nearly universal during isotretinoin therapy [47]. Musculoskeletal reactions such as arthralgia (2.36%) and myalgia (1.80%) are consistent with prior clinical reports [20,48]. Laboratory abnormalities included elevated triglycerides (2.34%), reflecting known isotretinoin-induced lipid alterations that may increase metabolic risk [49], and increased creatine phosphokinase (1.70%), which, although rare, may indicate muscle injury or rhabdomyolysis in the context of strenuous physical activity [50]. Ocular dryness (1.67%) was also reported, consistent with isotretinoin’s mucosal effects [47]. The distribution of these reactions in our dataset aligns with established safety profiles, underscoring the importance of monitoring gastrointestinal, metabolic, musculoskeletal, and mucocutaneous parameters throughout therapy.

Comparative analysis of neuropsychiatric versus non-neuropsychiatric cases revealed notable differences. Reports from non-EEA countries were significantly more frequent in the neuropsychiatric group (*p* < 0.001), possibly reflecting regional differences in prescribing practices, psychiatric care access, or reporting behavior. These differences may also be influenced by cultural stigma and sociocultural narratives surrounding mental health and acne. In some regions, stigma can discourage open disclosure of psychological symptoms, leading to underreporting, while in others, greater public awareness and openness to discussing emotional wellbeing may increase reporting. Evidence from sociocultural studies indicates that acne-related psychosocial distress is shaped by cultural beauty standards, gender norms, and perceived social judgment [51], all of which can impact the likelihood of reporting mental health symptoms in pharmacovigilance systems. Adolescents aged 12–17 years were also disproportionately affected (*p* < 0.001), reinforcing their susceptibility to psychiatric ADRs, as seen in FAERS data [52].

Importantly, reports from non-healthcare professionals, including patients and caregivers, were significantly more likely to contain neuropsychiatric symptoms (*p* < 0.0001). Logistic regression confirmed this association, showing that non-professional reporters had nearly double the odds of reporting such symptoms (*p* <0.001). This difference may partly reflect detection bias, as patients and caregivers are often more inclined to report subjective symptoms such as mood changes, whereas clinicians may underrecognize or underreport them, particularly when dermatological outcomes dominate clinical attention [53]. Nonetheless, this pattern also highlights the role of patient vigilance in early detection of subtle emotional or cognitive changes. This pattern is consistent with pharmacovigilance literature. First, patients emphasize subjective and quality-of-life impacts, mood change, anxiety, sleep disturbance, and emotional distress, whereas clinicians prioritize objective descriptors; consequently, patient reports more often capture psychosocial effects relevant to neuropsychiatry, while clinician reports lean toward traditional clinical details [54,55]. Second, reporting thresholds vary: Recent mixed-methods research and systematic reviews show that healthcare professionals consistently underreport because of time and workload constraints, uncertainty about causality, the belief that only serious or known reactions are worthy of being reported, laborious reporting procedures, and a lack of feedback to reporters. These factors can inhibit clinicians from reporting subjective psychiatric symptoms [56,57]. In contrast, patient reporting has expanded and can contribute to earlier signal detection, underscoring its complementary role in safety surveillance [58]. Third, health literacy and awareness have a significant impact on whether, when, and how patients report: While a lack of knowledge about pharmacovigilance calls for clearer communication and feedback loops, knowledge, beliefs about consequences, perceived roles in safety, and user-friendly digital channels all boost engagement [58,59].

The higher NPsR share among non-healthcare professional reporters in our data can be explained by a combination of these mechanisms, which also highlight the complementary value of patient reports in neuropsychiatric safety monitoring. These mechanisms include varying perceptions of psychiatric symptoms, a lower clinical reporting propensity for subjective events, and variable health literacy/awareness [58].

Age also emerged as a significant factor. Children (<12 years) and older adults (≥65 years) had lower odds of neuropsychiatric reporting compared to adolescents, potentially due to biological, perceptual, or social differences in symptom expression or detection [23,60]. Contrary to previous assumptions, sex was not a significant predictor of neuropsychiatric outcomes after adjustment, suggesting similar susceptibility in both sexes, a conclusion supported by earlier clinical research [61].

The model’s overall explanatory power was low (Nagelkerke R2 = 0.065), despite the fact that a number of factors achieved statistical significance. Therefore, these factors do not offer sufficient differentiation for individual-level prediction and only explain a small portion of the variability in whether an ICSR contains an NPsR. Our regression should be regarded as associational and hypothesis-generating rather than as a predictive risk-stratification tool, in accordance with established guidelines for interpreting spontaneous-report results [62,63].

### 3.2. Regulatory and Clinical Implications

Recent policy developments echo this emphasis. The UK’s Commission on Human Medicines (CHM) and MHRA advisory group recommended mandatory use of Patient-Reported Outcome Measures (PROMs) prior to initiating isotretinoin therapy, based on consistent feedback from patients and caregivers regarding early mood changes [64]. A 2023 MHRA follow-up report showed that 68% of stakeholders favored more rigorous psychiatric monitoring [65].

Regionally, reports from non-EEA countries remained slightly more likely to involve neuropsychiatric symptoms (*p* < 0.001), possibly reflecting awareness campaigns or differences in documentation standards [37,66,67]. Despite these insights, the logistic regression model explained only a small portion of the variability, highlighting the intrinsic limitations of spontaneous reporting systems. This low explained variance indicates that, although several predictors reached statistical significance, they capture only broad population-level trends and have limited utility for predicting whether a given individual report will involve neuropsychiatric symptoms. In light of the typically low pseudo-R2 found in the spontaneous-report model, these associations should guide population-level pharmacovigilance strategies (such as targeted monitoring and communication), but they should not be used to forecast neuropsychiatric events in individual patients or reports [62,63]. They remain valuable for generating hypotheses, informing risk stratification, and shaping future studies and pharmacovigilance protocols, particularly in high-risk populations such as adolescents and non-professionally reported cases.

Missing data on psychiatric history, acne severity, comorbidities, or concurrent medications constrain in-depth risk analysis [56,68,69].

This study offers one of the most comprehensive pharmacovigilance evaluations of isotretinoin to date, leveraging a large and longitudinal dataset of 33,381 individual case safety reports (ICSRs) submitted over a 20-year period to the EudraVigilance database. The extended observation window and high reporting volume enabled in-depth analysis of temporal fluctuations, demographic risk patterns, and reporting behavior across different regions and reporter types. The use of standardized MedDRA terminology and structured data allowed for detailed categorization of NPsRs, with depression and suicidal ideation emerging as the most frequently reported psychiatric effects. A major strength of the study is the incorporation of multivariable logistic regression to identify independent predictors of NPsR reporting. Statistically significant associations were detected for age group, sex, geographic origin, and, most prominently, reporter type. Reports submitted by non-healthcare professionals, such as patients and caregivers, had nearly twice the odds of including neuropsychiatric symptoms compared to those submitted by clinicians, highlighting the unique value of patient-reported outcomes in capturing subjective or underrecognized mental health effects. The regression findings were further validated through visual modeling of predicted logit probabilities, illustrating consistent directional trends across all variables. The large sample size, spanning diverse geographic settings, enhances the external validity and generalizability of the findings. Moreover, the temporal analysis captured significant shifts in reporting dynamics, most notably, the reporting spike in 2008 and the gradual decline in neuropsychiatric proportions after 2015, offering insight into the evolving landscape of isotretinoin safety surveillance.

Nonetheless, several limitations must be acknowledged. Like all studies based on spontaneous reporting systems, our analysis cannot establish causality or estimate absolute incidence rates. Reports merely document suspected associations observed in clinical practice and are inherently vulnerable to well-recognized biases, including both underreporting and selective reporting that may be influenced by media coverage, public awareness, or healthcare access. Another fundamental limitation is the absence of denominator data, such as the total number of isotretinoin-exposed patients during the study period, which prevents the calculation of absolute risk estimates or direct comparison with background rates in the general population. In addition, the variability in MedDRA coding poses challenges for standardizing symptom definitions. Conceptually similar terms (e.g., “depression” vs. “depressed mood”) may be used inconsistently by different reporters, and even after grouping, some heterogeneity inevitably persists. While we applied rigorous data-cleaning procedures, such variability cannot be fully eliminated. Missing data also represented an obstacle: Age was not specified in more than a quarter of reports (26.7%), which limited the granularity of subgroup analyses. Equally important, the dataset did not capture essential clinical covariates such as acne severity, treatment duration, prior psychiatric history, or concurrent medications. This lack of contextual information restricts the capacity to disentangle whether psychiatric symptoms reflect isotretinoin exposure per se, the psychosocial burden of acne, or underlying psychiatric vulnerability. Finally, while our multivariable model identified statistically significant predictors of neuropsychiatric reporting, the explained variance was modest (Nagelkerke R^2^ = 0.065). This result is not unexpected, given the constraints of pharmacovigilance data, but it underscores that spontaneous reporting systems should be interpreted as hypothesis-generating rather than confirmatory.

These results should thus be interpreted as exploratory and hypothesis-generating, underscoring the need for prospective studies and targeted pharmacoepidemiologic research to further elucidate the neuropsychiatric safety profile of isotretinoin, particularly in high-risk subgroups. Taken together, these findings contribute to a more nuanced understanding of isotretinoin’s neuropsychiatric safety in real-world settings and emphasize the importance of continued pharmacovigilance, structured psychiatric monitoring, and patient-centered care as integral components of treatment planning.

## 4. Materials and Methods

### 4.1. Data Source and Study Design

**Figure 4:**
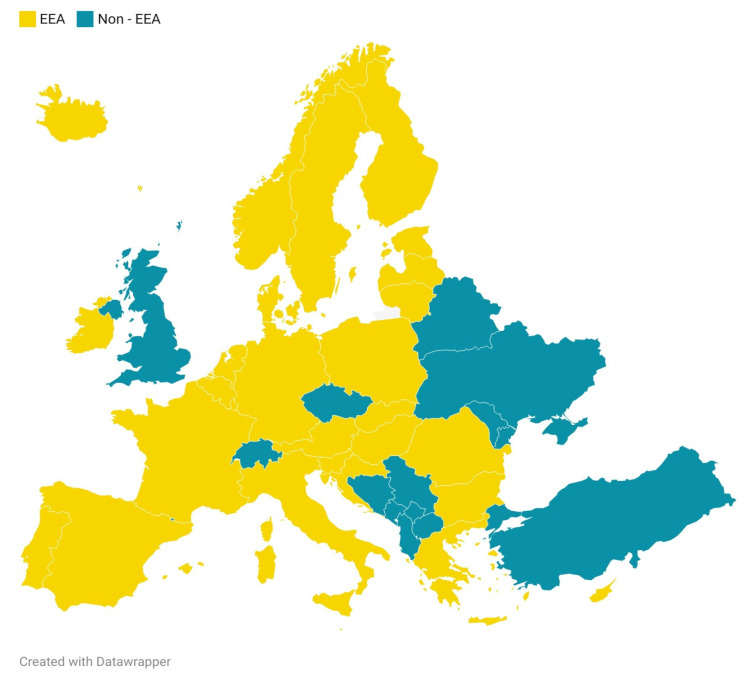
Geographical distribution of reports across European Economic Area (EEA) and non-EEA countries. Created by the authors with Datawrapper API (v3).

We conducted a retrospective analysis of individual case safety reports (ICSRs) submitted to EudraVigilance, the European Union’s pharmacovigilance database managed by the European Medicines Agency (EMA). The study period extended from 1 January, 2005, through 28 April, 2025. All spontaneous reports listing isotretinoin as a suspected or interacting medication were extracted in comma-separated value (CSV) format (data cutoff: 6 April, 2025). The reports were obtained from the publicly accessible ADRreports portal [70], which provides anonymized pharmacovigilance data submitted by healthcare professionals and non-professionals from both the European Economic Area (EEA) and non-EEA regions. In accordance with the EMA’s classification, the EEA comprises all European Union (EU) Member States plus Iceland, Liechtenstein, and Norway. Reports from countries outside this group were classified as non-EEA. For the period after the United Kingdom’s withdrawal from the EU (Brexit), UK-origin reports were counted as non-EEA (Figure 4).

**Figure 5:**
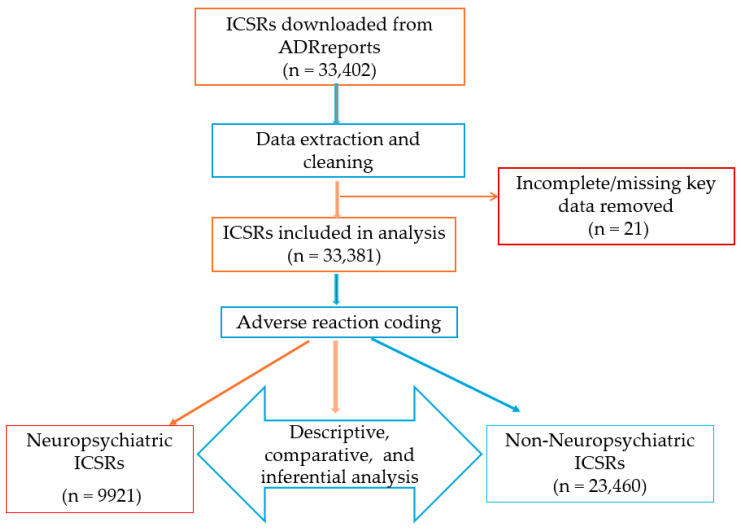
Case selection process and analysis workflow.

An initial total of 33,402 ICSRs involving isotretinoin were retrieved. After removing 21 duplicate or erroneous entries, the final dataset included 33,381 valid reports. These formed the basis for all descriptive, comparative, and inferential analyses. The study workflow was structured in four stages: data extraction and cleaning, adverse reaction coding, descriptive and comparative statistical analysis, and multivariable modeling (Figure 5).

### 4.2. Data Extraction

Annual datasets were downloaded from the ADRreports portal and merged into a unified master file. Each report included standardized variables such as case ID, submission year, patient sex and age group (using EMA categories), country of origin, reporter type (healthcare or non-healthcare professional), suspected drug(s), interaction data, and a list of adverse reactions coded using MedDRA preferred terms (PTs). Neuropsychiatric reactions were identified using predefined PTs mapped to relevant MedDRA system organ classes. For the complementary analysis of non-neuropsychiatric adverse reactions, all clinically relevant preferred terms (PTs) were retained. Related terms referring to the same medical condition (e.g., ulcerative colitis, Crohn’s disease, and inflammatory bowel disease) were grouped into broader categories to enhance interpretability. All statistical analyses were conducted using this consolidated dataset.

### 4.3. Adverse Reaction Coding

All adverse reactions were coded using MedDRA PT terminology [71]. In the raw dataset, multiple reactions could be listed per case within a single text field, each comprising the PT name, outcome (e.g., recovered, not recovered), and seriousness criteria. Neuropsychiatric adverse reactions were defined a priori as any PT classified under either of the MedDRA system organ classes (SOCs): psychiatric disorders or nervous system disorders [72,73]. This operational definition allowed for the inclusion of a wide range of clinically relevant symptoms, both psychological (e.g., depression, anxiety, suicidal ideation, suicide attempt) and neurological (e.g., headache, seizures, dizziness), that are potentially attributable to isotretinoin. While suicidal ideation and suicide attempt are coded as separate PTs in MedDRA, reflecting the difference between reported thoughts of self-harm and actual self-injurious behavior, both were retained as distinct entries within our dataset to capture the full spectrum of suicide-related reporting. MedDRA’s hierarchical structure assigns each PT a primary SOC and, when relevant, one or more secondary SOCs. According to MedDRA guidance, terms with etiological relevance to the central nervous system are typically grouped under nervous system disorders (primary) and may also appear under psychiatric disorders (secondary), ensuring comprehensive coverage of neuropsychiatric symptomatology [73].

To ensure a precise classification, each PT reported in the dataset was checked against an official MedDRA list of terms belonging to these two SOCs. Filtering and classification of neuropsychiatric PTs were conducted using semi-automated string-based functions in Microsoft Excel (version 2506). Functions such as SEARCH, IF, and FILTER were applied to detect key terms or partial matches across reaction descriptions. Only PTs that were actually reported in the dataset and matched the two MedDRA SOCs were retained. Each case was then classified as neuropsychiatric (Yes/No) based on whether at least one such PT was identified. All classification steps were double-checked for consistency and correctness. 

### 4.4. Statistical Analysis

Descriptive statistics were used to summarize annual trends, demographic distributions (sex and age), reporter types, and geographic origin for the full dataset and the subset involving neuropsychiatric reactions. Frequencies and percentages of the most frequently reported neuropsychiatric PTs were also calculated. Initial data cleaning, tabulation, and descriptive summaries were performed in Microsoft Excel (version 2566), while inferential statistical analyses were conducted in JASP (version 0.19.3.0, an open-source statistical software.Comparative analyses between NPsR and non-NPsR cases were conducted using chi-square tests across categorical variables. To identify independent predictors of neuropsychiatric reporting, we constructed a multivariable logistic regression model with a binary outcome (presence vs. absence of NPsRs). Predictor variables included age group, sex, reporter type, and geographic origin (EEA vs. non-EEA). For regression purposes, age was categorized into five broader groups: <12 years, 12–17 years, 18–64 years, ≥65 years, and NSp. The <12 years group combined infants aged 0–1 month, children aged 2 months–2 years, and those aged 3–11 years, while the ≥65 years group included all older adults. This aggregation was applied only in the regression model to ensure sufficient cell counts, avoid sparse-data bias, and improve model stability, given the very low frequency of isotretinoin exposure in the youngest and oldest age categories. Odds ratios (ORs) and 95% confidence intervals (CIs) were calculated, with significance set at *p* < 0.05 (two-tailed).

Because the study relied exclusively on anonymized public pharmacovigilance data, no patient consent or ethics committee approval was required. All analyses adhered to the EMA’s public data usage policies.

## 5. Conclusions

This 20-year pharmacovigilance analysis of isotretinoin-related adverse event reports from the EudraVigilance database reveals a persistent and clinically relevant signal of neuropsychiatric symptoms, particularly depression and suicidal ideation, across a large and demographically diverse patient population. Nearly one-third of all reports included at least one neuropsychiatric reaction, with higher reporting rates observed among adolescents, non-healthcare reporters, and cases originating from outside the European Economic Area.

Although causality cannot be inferred from spontaneous reporting data, the observed demographic and contextual patterns highlight subgroups that warrant increased clinical attention. These findings underscore the importance of integrating structured mental health screening and ongoing psychiatric monitoring into isotretinoin treatment protocols, especially for younger patients and those with pre-existing psychosocial vulnerabilities. Closer collaboration between dermatologists, patients, and mental health professionals may enhance both the safety and the therapeutic effectiveness of isotretinoin in routine clinical practice.

## Figures and Tables

**Table 1:** Demographic, reporting, and geographic characteristics of isotretinoin-related ICSRs (2005–2025).

Category	Value	Total Cases	Frequency (%)
Sex	Female	16,806	50.4
	Male	14,670	43.9
	Not specified	1905	5.7
Age group	0–1 month	152	0.5
	2 months–2 years	69	0.2
	3–11 years	174	0.5
	12–17 years	7512	22.5
	18–64 years	16,428	49.2
	65–85 years	127	0.4
	More than 85 years	3	0.01
	Not specified	8919	26.7
Reporter type	Healthcare professional	19,596	58.7
	Non-healthcare professional	13,690	41.0
	Not specified	95	0.3
Geographic origin	European Economic Area	12,494	37.4
	Non-European Economic Area	20,886	62.6
	Not specified	1	0.003

**Table 2:** Annual distribution of neuropsychiatric and non-neuropsychiatric adverse reactions to isotretinoin (2005–2025).

Year	Non-NPsRs	%	NPsRs	%
2005	629	85.230	109	14.770
2006	793	84.093	150	15.907
2007	780	79.673	199	20.327
2008	5560	67.987	2618	32.013
2009	702	79.863	177	20.137
2010	787	78.494	216	21.506
2011	1192	64.989	641	35.011
2012	1469	58.312	1050	41.688
2013	1665	59.986	1109	40.014
2014	1382	65.969	712	34.031
2015	855	65.703	446	34.297
2016	556	66.147	285	33.853
2017	559	74.733	189	25.267
2018	773	76.971	232	23.029
2019	951	74.886	319	25.114
2020	791	75.984	250	24.016
2021	750	77.571	217	22.429
2022	1514	87.339	219	12.661
2023	705	76.033	222	23.967
2024	1072	77.840	305	22.160
2025	205	76.975	61	23.025

NPsR = neuropsychiatric adverse reaction.

**Table 3:** Top 10 most frequently reported neuropsychiatric adverse reactions (NPsRs) to isotretinoin, 2005–2025.

Reaction	Frequency	% of NPsRs	% of NP Cases
Depression	4967	30.67	50.72
Suicidal ideation	1348	8.32	13.76
Headache	1200	7.41	12.25
Anxiety	1092	6.74	11.15
Emotional distress	780	4.82	7.96
Suicide attempt	445	2.75	4.54
Depressed mood	346	2.14	3.53
Completed suicide	335	2.07	3.42
Dizziness	314	1.94	3.21
Seizure	281	1.73	2.87

NPsR = neuropsychiatric adverse reaction.

**Table 4:** Top reported neuropsychiatric adverse reactions to isotretinoin by age and sex group, 2005–2025.

Reaction	Sex	0–1 Month 0–2 n (%)	12–17 Years n (%)	18–64 Years n (%)	3–11 Years n (%)	65–85 Years n (%)	>85 Years n (%)	Not Specified n (%)
Depression	Female	-	483 (37.4)	1641 (58.1)	7 (63.6)	4 (44.4)	1 (100.0)	413 (49.9)
	Male	-	806 (62.3)	1176 (41.6)	4 (36.4)	5 (55.6)	-	351 (42.4)
	NSp	-	4 (0.3)	9 (0.3)	-	-	-	63 (7.6)
Suicidal ideation	Female	-	115 (29.5)	364 (54.1)	2 (100.0)	-	-	128 (45.7)
	Male	-	272 (69.7)	307 (45.6)	-	3 (100.0)	-	104 (37.1)
	NSp	-	3 (0.8)	2 (0.3)	-	-	-	48 (17.1)
Headache	Female	-	146 (43.8)	457 (68.2)	6 (54.5)	-	-	93 (50.3)
	Male	-	187 (56.2)	209 (31.2)	5 (45.5)	1 (100.0)	-	57 (30.8)
	NSp	-	-	4 (0.6)	-	-	-	35 (18.9)
Anxiety	Female	1 (100.0)	95 (33.8)	374 (57.9)	2 (100.0)	-	-	63 (39.4)
	Male	-	184 (65.5)	270 (41.8)	-	2 (100.0)	-	83 (51.9)
	NSp	-	2 (0.7)	2 (0.3)	-	-	-	14 (8.8)
Emotional distress	Female	-	59 (28.4)	232 (46.3)	1 (20.0)	1 (50.0)	-	27 (42.2)
	Male	-	149 (71.6)	267 (53.3)	4 (80.0)	1 (50.0)	-	33 (51.6)
	NSp	-	-	2 (0.4)	-	-	-	4 (6.2)
Suicide attempt	Female	-	81 (40.5)	70 (46.7)	1 (100.0)	-	-	41 (43.6)
	Male	-	117 (58.5)	78 (52.0)	-	-	-	38 (40.4)
	NSp	-	2 (1.0)	2 (1.3)	-	-	-	15 (16.0)
Depressed mood	Female	-	36 (35.6)	103 (55.7)	-	-	-	26 (44.8)
	Male	-	65 (64.4)	79 (42.7)	-	2 (100.0)	-	22 (37.9)
	NSp	-	-	3 (1.6)	-	-	-	10 (17.2)
Completed suicide	Female	-	16 (18.0)	31 (18.9)	-	-	-	12 (14.6)
	Male	-	72 (80.9)	131 (79.9)	-	-	-	55 (67.1)
	NSp	-	1 (1.1)	2 (1.2)	-	-	-	15 (18.3)
Dizziness	Female	-	39 (45.3)	118 (65.6)	-	1 (25.0)	-	30 (68.2)
	Male	-	47 (54.7)	62 (34.4)	-	3 (75.0)	-	10 (22.7)
	NSp	-	-	-	-	-	-	4 (9.1)
Seizure	Female	-	26 (27.1)	55 (47.4)	1 (50.0)	1 (100.0)	-	24 (36.4)
	Male	-	66 (68.8)	60 (51.7)	1 (50.0)	-	-	19 (28.8)
	NSp	-	4 (4.2)	1 (0.9)	-	-	-	23 (34.8)
Suicide attempt	Female	-	81 (40.5)	70 (46.7)	1 (100.0)	-	-	41 (43.6)
	Male	-	117 (58.5)	78 (52.0)	-	-	-	38 (40.4)
	NSp	-	2 (1.0)	2 (1.3)	-	-	-	15 (16.0)
Depressed mood	Female	-	36 (35.6)	103 (55.7)	-	-	-	26 (44.8)
	Male	-	65 (64.4)	79 (42.7)	-	2 (100.0)	-	22 (37.9)
	NSp	-	0 (0.0)	3 (1.6)	-	-	-	10 (17.2)
Completed suicide	Female	-	16 (18.0)	31 (18.9)	-	-	-	12 (14.6)
	Male	-	72 (80.9)	131 (79.9)	-	-	-	55 (67.1)
	NSp	-	1 (1.1)	2 (1.2)	-	-	-	15 (18.3)
Dizziness	Female	-	39 (45.3)	118 (65.6)	-	1 (25.0)	-	30 (68.2)
	Male	-	47 (54.7)	62 (34.4)	-	3 (75.0)	-	10 (22.7)
	NSp	-	-	-	-	-	-	4 (9.1)
Seizure	Female	-	26 (27.1)	55 (47.4)	1 (50.0)	1 (100.0)	-	24 (36.4)
	Male	-	66 (68.8)	60 (51.7)	1 (50.0)	-	-	19 (28.8)
	NSp	-	4 (4.2)	1 (0.9)	-	-	-	23 (34.8)

NSp = not specified.

**Table 5:** Top 10 most frequently reported non-neuropsychiatric adverse reactions (NPsRs) to isotretinoin, 2005–2025.

Reaction	Count	% of Non-NPsR Cases
Inflammatory bowel disease	3642	15.44
Dry skin	792	3.36
Lip dry	629	2.67
Arthralgia	557	2.36
Blood triglycerides increased	553	2.34
Irritable bowel syndrome	526	2.23
Myalgia	424	1.8
Acne	409	1.73
Blood creatine phosphokinase increased	401	1.7
Dry eye	393	1.67

NPsR = neuropsychiatric adverse reaction.

**Table 6:** Distribution of neuropsychiatric adverse reactions by demographic and reporting characteristics, *p* < 0.001.

Category	Observed_No	Observed_Yes	% Non-NPsRs	% NPsRs	Chi^2^
Sex					202.90
Female	12,022	4784	71.5	28.1	
Male	9948	4822	67.4	32.6	
NSp	1618	287	84.9	15.1	
Age group					646.51
0–1 month	145	7	95.4	4.6	
2 months–2 years	65	4	94.2	5.8	
3–11 years	141	33	81.0	19.0	
12–17 years	4785	2727	63.7	36.3	
18–64 years	11,141	5287	67.8	32.2	
65–85 years	95	29	76.6	23.4	
>85 years	2	1	66.7	33.3	
NSp	7264	1655	81.4	18.6	
Reporter type					768.45
Healthcare professional	14,900	4696	76.0	24.0	
Non-healthcare professional	8360	5330	61.1	38.9	
NSp	328	92	78.1	21.9	
Region					83.74
European Economic Area	9203	3291	73.6	26.4	
Non-European Economic Area	14,343	6543	68.7	31.3	
NSp	42	9	82.4	17.6	

NSp = not specified; NPsR = neuropsychiatric adverse reaction.

**Table 7:** Logistic regression predicting the likelihood of neuropsychiatric adverse reactions.

Predictor Category	Category	Estimate	Std. Error	OR (95% CI)	z	Wald	*p*-Value
Age group	12–17 years (Ref)	-	-	-	-	-	-
	18–64 years	−0.204	0.03	0.82 (0.77–0.87)	−6.713	45.059	<0.001
	<12 years	−1.455	0.163	0.23 (0.17–0.32)	−8.944	79.992	<0.001
	NSp	−0.814	0.039	0.44 (0.41–0.48)	−21.116	445.891	<0.001
	>65 years	−0.551	0.212	0.58 (0.38–0.87)	−2.596	6.739	0.009
Sex	Female (Ref)	-	-	-	-	-	-
	Male	0.107	0.026	1.11 (1.06–1.17)	4.154	17.26	<0.001
	NSp	−0.258	0.069	0.77 (0.67–0.88)	−3.735	13.954	<0.001
Reporter type	Healthcare professional (Ref)	-	-	-	-	-	-
	Non-healthcare professional	0.605	0.026	1.83 (1.74–1.93)	23.449	549.835	<0.001
	NSp	−0.26	0.265	0.77 (0.46–1.30)	−0.979	0.958	0.328
	EEA (Ref)	-	-	-	-	-	-
	Non-EEA	0.152	0.027	1.16 (1.10–1.23)	5.649	31.916	<0.001

Note: OR = odds ratio; CI = confidence interval; EEA = European Economic Area; NSp = not specified. Reference categories are as follows: age group = 12–17 years, sex = female, reporter = healthcare professional, region = EEA, and age group = 12–17 years. The binary outcome was the presence (Yes) vs. absence (No) of at least one neuropsychiatric adverse reaction.

## Data Availability

The original contributions presented in the study are included in the article; further inquiries can be directed to the corresponding authors.

## Abbreviations

The following abbreviations are used in this manuscript:

ADRs	Adverse drug reactions
CHM	Commission on Human Medicines
CI	Confidence interval
CNS	Central nervous system
EEA	European Economic Area
EMA	European Medicines Agency
FAERS	FDA Adverse Event Reporting System
FDA	Food and Drug Administration
ICH	International Council for Harmonization
ICSRs	Individual case safety reports
MedDRA	Medical Dictionary for Regulatory Activities
MHRA	Medicines and Healthcare products Regulatory Agency
NPsR(s)	Neuropsychiatric adverse reaction(s)
OR	Odds ratio
PROs	Patient-reported outcomes
PROMs	Patient-reported outcome measures
PT(s)	Preferred term(s)
SOC	System organ class
UK	United Kingdom
US / U.S.	United States
